# Myoblast-derived exosomal Prrx2 attenuates osteoporosis via transcriptional regulation of lncRNA-MIR22HG to activate Hippo pathway

**DOI:** 10.1186/s10020-023-00649-y

**Published:** 2023-04-20

**Authors:** Yunchao Li, Xiaoxiao Wang, Changyu Pan, Hui Yuan, Xinyi Li, Zejun Chen, Haoyu He

**Affiliations:** Department of Spine Surgery, The Second Xiangya Hospital of Central South University, No. 139, RenMin Middle Road, Changsha, 410001 Hunan Province P.R. China

**Keywords:** Sarcopenia, Osteoporosis, Osteogenic differentiation, Prrx2, MIR22HG, YAP signaling pathway

## Abstract

**Background:**

Sarcopenia and osteoporosis are common diseases that predominantly affect older individuals. The interaction between muscle and skeleton exerts pivotal roles in bone remodeling. This study aimed to explore the function of myoblast-derived exosomal Prrx2 in osteogenic differentiation and its potential mechanisms.

**Methods:**

Exosomes were isolated from myogenic differentiated C2C12 cells. qRT-PCR and Western blotting were used to determine target molecule expression. Osteogenic differentiation of BMSCs was evaluated by Alizarin red staining, ALP activity and levels of OCN, OPN, RUNX2, and BMP2. Dual-luciferase reporter assay, RIP, and ChIP assays were performed to verify the interaction between molecules. The nuclear translocation of YAP1 was observed by immunofluorescence staining. In vivo osteoporotic model was established by ovariectomy in mice. Bone loss was examined using HE staining.

**Results:**

Prrx2 expression was elevated in myogenic differentiated C2C12 cells and their exosomes. Myoblast-derived exosomal Prrx2 enhanced osteogenic differentiation of BMSCs. Delivering exosomal Prrx2 directly bond to MIR22HG promoter and promoted its transcription and expression. MIR22HG enhanced expression and nuclear translocation of YAP via sponging miR-128, thus facilitating BMSC osteogenic differentiation. Knockdown of exosomal Prrx2 suppressed osteogenic differentiation, which could be abolished by MIR22HG overexpression. Similarly, miR-128 inhibitor or YAP overexpression reversed the inhibitory effect of MIR22HG depletion or miR-128 mimics on osteogenic differentiation. Finally, myoblast-derived exosomal Prrx2 alleviated osteoporosis in mice via up-regulating MIR22HG and activating the Hippo pathway.

**Conclusion:**

Myoblast-derived exosomal Prrx2 contributes to transcriptional activation of MIR22HG to activate YAP pathway via sponging miR-128, thereby facilitating osteogenic differentiation of BMSCs.

**Supplementary Information:**

The online version contains supplementary material available at 10.1186/s10020-023-00649-y.

## Background

The coexistence of sarcopenia and osteoporosis frequently occurred in seniors has been recognized as a syndrome called ‘osteosarcopenia’. Osteoporosis, characterized by declined bone mineral density, has closely association with fracture (Kanis 1994). Sarcopenia is a musculoskeletal disease that is defined as a remarkable decrease in muscle strength, muscle mass and function (Cao and Morley 2016). As is reported, a gradual decrease in bone mineral density (about 1-1.5% per year) and muscle mass (about 1% per year) is found in people more than 60 years of age (Daly et al. 2013), which may increase risk of osteosarcopenia. It has been recognized that bone and muscle closely interact with each other (Brotto and Bonewald 2015), and there is a structural and functional degeneration of them with age (Locquet et al. 2019). Recent studies have suggested that the interaction between bone and muscle is achieved by endocrine (Girgis 2015). A lot of regulators derived from myoblast or bone play crucial roles in the cross-talk between bone and muscle (Guo et al. 2017; Hamrick 2017).

Exosomes are a type of extracellular vesicles secreted by different kinds of cells, including myoblasts. Guescini et al., firstly reported the release of exosomes by myoblasts (Guescini et al. 2010). It has been discovered that exosomes are responsible for cell-cell communication, which exert pivotal roles in various biological processes via delivering bioactive molecules to recipient cells (Camussi et al. 2010). For instance, myoblast C2C12 -derived exosomes was demonstrated to improve endothelial cell function (Nie et al. 2019). Fulzele et al., revealed that circulating miR-34a level in muscle-derived exosomes was increased with age, which resulted in senescence of bone marrow stem cells (Fulzele et al. 2019). Due to the contribution of exosomes to intercellular communication, exosomes have been recognized as a promising strategy for treating multiple disorders, including sarcopenia (Rong et al. 2020). It was remarkable that exosomes released from C2C12 myoblasts could promote osteogenic differentiation via transferring miR-27a-3p (Xu et al. 2018). These findings suggest that myoblast-derived exosomes may take part in the regulation of osteogenesis during osteoporosis.

Paired-related homeobox 2 (Prrx2) is a transcription factor belonged to the paired-related homeobox protein family. A higher expression of Prrx2 in mesenchymal tissues during embryonic development plays key roles in craniofacial and limb organogenesis (Lu et al. 1999; Norris and Kern 2001; ten Berge et al. 1998). Double mutant of Prrx1 and Prrx2 resulted in defective skeletogenesis in mice (ten Berge et al. 1998). A previous study by Norris et al., investigated the transcription regulatory regions of Prrx2 in NIH 3T3 cells and C2C12 cells (Norris and Kern 2001). Interestingly, our preliminary experimental results indicated that Prrx2 expression was increased in C2C12 myoblast-derived exosomes. However, whether exosomal Prrx2 from C2C12 myoblasts can promote osteogenic differentiation and alleviate osteoporosis remains unclear, which deserves further investigation.

Long non-coding RNAs (lncRNAs) are defined as non-protein-coding RNA transcripts comprised of more than 200 nucleotides. Mounting evidence has demonstrated that lncRNAs are pivotal modulators in various pathophysiological processes including osteoporosis (Chen et al. 2020; Yang et al. 2019). A series of lncRNAs have been identified as regulators for osteogenic differentiation. For instance, Zhu et al., reported that lncRNA-NEAT1 promoted renal interstitial fibroblast osteogenic differentiation via enhancing BMP2 expression (Zhu et al. 2021). LncRNA-MIR22 host gene (MIR22HG), located on chromosome 17p13.3, has been revealed as a crucial regulator for multiple malignancies (Deng et al. 2021; Jin et al. 2021). MIR22HG has also been documented to facilitate osteogenic differentiation of BMSCs through modulating PTEN/AKT pathway (Jin et al. 2020). Notably, JASPAR database predicted that there were binding sites of Prrx2 in MIR22HG promoter. Therefore, we speculated that myoblast-derived exosomal Prrx2 might regulate osteogenic differentiation via transcriptional regulating MIR22HG.

In the present work, we verified our hypothesis and found that Prrx2 was up-regulated in the myoblast-derived exosomes, which facilitated BMSC osteogenic differentiation and relieved osteoporosis. Mechanistically, exosomal Prrx2 promoted transcription and expression of MIR22HG, and thereby activated Hippo signaling pathway via sponging miR-128 to increase YAP1 expression. Overall, our observations provide a new strategy for treating osteoporosis.

## Methods

### Cell culture and treatment

C2C12 myoblasts were purchased from American Type Culture Collection and maintained in DMEM (Thermo Fisher, Waltham, MA, USA) containing 10% FBS (Thermo Fisher) at 37 °C with 5% CO_2_. To induce myogenic differentiation, C2C12 cells were cultured in DMEM supplemented with 2% horse serum (Thermo Fisher) for 1 d, 4 d or 7 d.

### Exosome isolation and identification

Exosomes were isolated from the supernatant of myogenic differentiated C2C12 cells after incubation in serum-free DMEM for 24 h. Briefly, the supernatant was subjected to centrifugation (10,000 g, 30 min) at 4 °C and filtration using a 0.22 μm pore size. After ultra-centrifugation (100,000 g, 70 min) at 4 °C twice, the exosome pellets were resuspended in PBS and observed under a transmission electron microscopy (Thermo Fisher). To further identify the separated exosomes, the expression of CD63, CD9, TSG101, HSP70, and Calnexin was evaluated by Western blotting. The size of exosomes was evaluated using Nanoparticle tracking analysis.

### Isolation of bone marrow mesenchymal stem cells (BMSCs) and identification

BMSCs were isolated from mice as previously described(Du et al. 2021). In brief, the bone marrow was collected from the femurs and tibiae of mice and cut into 1–2 mm cuts. After digestion with collagenase II and IV for 45 min, the digested cells were cultured in proliferation medium (PM): α-MEM (Thermo Fisher) supplemented with 10% FBS. In addition, the isolated BMSCs were identified using flow cytometry analysis. To achieve this, BMSCs were probed with PE-conjugated antibodies against CD105, CD90, CD73, CD45 and CD34 (BD Pharmingen, Franklin Lake, NJ, USA) for 30 min and then detected on a flow cytometer (Partec, Nuremberg, Germany).

For the induction of osteogenic differentiation, the 100% confluent BMSCs were maintained in osteoblastic medium (OM) containing 10% FBS, 0.2 mM ascorbic acid, 10 mM β-glycerophosphate, and 100 nM dexamethasone.

### Cellular uptake of exosomes

To determine the localization of exosomes in recipient cells, exosomes were subjected to PKH26 labelling (Sigma, Saint Louis, MO, USA). Then BMSCs were incubated with PKH26-labelled exosomes at 37 °C for 24 h. After nuclear staining with DAPI, localization of exosomes in BMSCs was observed under a fluorescence microscopy (Olympus, Tokyo, Japan).

### Lentivirus infection or cell transfection

Lentiviruses containing vector, MIR22HG overexpressing plasmid, negative control shRNA (shNC), shMIR22HG, shPrrx-2, inhibitor NC, miR-128 inhibitor, mimics NC, miR-128 mimics, YAP1 overexpressing plasmid were provided by GeneChem (Shanghai, China). BMSCs were infected with the above lentiviruses at a multiplicity of infection in the presence of 5 µg/mL polybrene according to a previous study (Yang et al. 2021). Transfection was carried out by using Lipofectamine 3000 (Invitrogen, Missouri, USA) according to manufacturer’s protocol. Cells were collected for further analysis 48 h after transfection. The stably transfected cells were selected using G418.

### ALP activity detection

After the induction of osteogenic differentiation for 7 d or 14 d, ALP activity of BMSCs was assessed using the ALP assay kit (Nanjing Jiancheng Bioengineering Institute, China) according to the manufacturer’s protocol.

### Alizarin red staining

The calcium deposition of BMSCs after cultured in OM for 7 d or 14 d was evaluated by Alizarin Red staining. Briefly, BMSCs were fixed in 4% paraformaldehyde for 20 min and washed by PBS for three times. Then BMSCs were stained with Alizarin red solution (Sigma Aldrich, Saint Louis, MO, USA) and photographed under a microscope (Olympus, Tokyo, Japan).

### Chromatin immunoprecipitation (ChIP) assay

The binding of Prrx-2 to the promoter of MIR22HG was determined by ChIP assay using the EZ-ChIP™ Kit (Millipore, Billerica, MA, USA) according to the protocol. Cells were incubated with formaldehyde to obtain DNA-protein crosslinks. After DNA fragmentation by sonicating, the samples were immunoprecipitated with Prrx-2 (1:50, Santa Cruz) or IgG (1:50, Abcam) antibody. Finally, the precipitated chromatin was subjected to qRT-PCR.

### Dual luciferase reporter assay

The MIR22HG promoter regions containing Prrx-2 binding sites were inserted into the pGL3-basic vector. Besides, the wild type (WT) or mutant (MUT) sequences of MIR22HG/YAP1 containing the miR-128 binding sites were cloned into the psiCHECK2 vector. After transfection with the established reporter vector together with or without miR-128 mimics/mimics NC using Lipofectamine 2000 (Thermo Fisher) for 48 h, the luciferase activity was measured using the Dual-Luciferase Reporter Assay System (Promega).

**RNA immunoprecipitation (RIP) assay**The interaction between MIR22HG and miR-128 was validated by RIP assay using the EZ-Magna RIP Kit (Millipore). In brief, cell lysates of BMSCs were obtained after lysing in RIP lysis buffer. The lysates were immunoprecipitated with magnetic beads conjugated with AGO2 (1: 50, Abcam) or IgG (1:50, Abcam) at 4 °C overnight. Thereafter, the immunoprecipitated RNA was extracted, purified and detected by qRT-PCR.

### Immunofluorescence staining

For immunofluorescence staining, BMSCs were fixed in 4% paraformaldehyde and permeated with 0.1% Triton X-100. After blocking in 2% BSA, BMSCs were probed with YAP1 (1:500, Abcam) at 4 °C overnight. Then incubation with Goat Anti-Rabbit IgG H&L (1:500, Abcam) for 1 h at room temperature was carried out. After nuclear staining with the 4′,6-diamidino-2-phenylindole (DAPI), the nuclear translocation of YAP1 was observed under a fluorescence microscope.

### Mouse model of osteoporosis

Six-week-old female C57BL/6 J mice were purchased from SJA Laboratory Animal Co., Ltd (Hunan, China) and randomly divided into four groups (n = 6 per group): sham group, ovariectomized (OVX) group, OVX + Exo-shNC group, OVX + Exo-shPrrx-2 group. To induce osteoporosis, the mice in OVX group were subjected to bilateral ovariectomy. The sham mice were received lipectomy near the ovary. Four weeks after bilateral ovariectomy, the osteoporotic model was successfully established. The mice in OVX + Exo-shNC group or OVX + Exo-shPrrx-2 group were injected with exosomes derived from myogenic differentiated C2C12 cells that were transfected with shNC or shPrrx-2 via the femoral bone marrow periosteum twice a week (Zhang et al. 2020). Two weeks after the first injection, all mice were euthanized by an over dose of sodium pentobarbital and the femurs were collected. The bone volume per tissue volume (BV/TV), trabecular number (Tb.N) and trabecular thickness (Tb.Th) were measured using micro-CT.

### Hematoxylin/eosin (HE) staining

The collected femurs were fixed in 4% paraformaldehyde and then incubated in 15% EDTA for 21 d for decalcification, followed by paraffin embedding. The femurs were cut into 5 μm-sections and stained with a HE Staining Kit (Solarbio, Beijing, China). The photographs were taken under a light microscope.

### Quantitative reverse transcription-polymerase chain reaction (qRT-PCR)

Total RNA was isolated using Trizol reagent (Thermo Fisher). Subsequently, the RNA was converted into cDNA using the PrimeScript RT Reagent Kit (Takara, Tokyo, Japan) for mRNAs/lncRNA, and the miScript II RT kit (Qiagen, Dusseldorf, Germany) for miR-128, respectively. Quantitative real-time PCR was performed using the SYBR green PCR kit (Roche, Basel, Switzerland). Relative transcript levels normalized to GAPDH or U6 were calculated with the 2^–ΔΔCt^ method. Table 1 lists the primer sequences.

### Western blotting

Total protein was extracted using the Whole Cell Lysis Assay kit (KeyGEN, Nanjing, China) and quantified using the BCA Protein Quantitation Assay kit (KeyGEN). Protein samples were subjected to sodium dodecyl sulfate gel electrophoresis and transferred to a polyvinylidene fluoride membranes. Subsequently, the membranes were incubated with 5% skimmed milk for 1 h, and probed with primary antibodies against MyoD (1:1000, Abcam, Cambridge, UK), MyHC (1:1000, Abcam), MyoG (1:1000, Abcam), Prrx-2 (1:1000, Abcam), CD63 (1:1000, Abcam), CD9 (1:1000, Cell Signaling Technology, Trask Lane Danvers, MA, USA), TSG101 (1:1000, Abcam), HSP70 (1:1000, Abcam), Calnexin (1:1000, Abcam), yes-associated protein 1 (YAP1, 1:1000, Abcam), OCN (1:2000, Abcam), OPN (1:1000, Abcam), RUNX2 (1:1000, Abcam), BMP2 (1:1000, Abcam) and GAPDH (1:1000, Abcam) at 4 °C overnight. After incubation with the appropriate secondary antibody, the protein bands were visualized using the ECL Detection Kit (KeyGEN).

### Statistical analysis

Data are shown as mean ± standard deviation (SD) and analyzed using SPSS17.0 software. Student t-test was used for statistical analysis between two experimental groups, while One-Way ANOVA followed by Tukey post hoc test was adopted to compare data from more than two groups. p < 0.05 was considered as statistically significant.

## Results

### Prrx-2 is enriched in exosomes derived from myogenic differentiated C2C12 cells

**Fig. 1:**
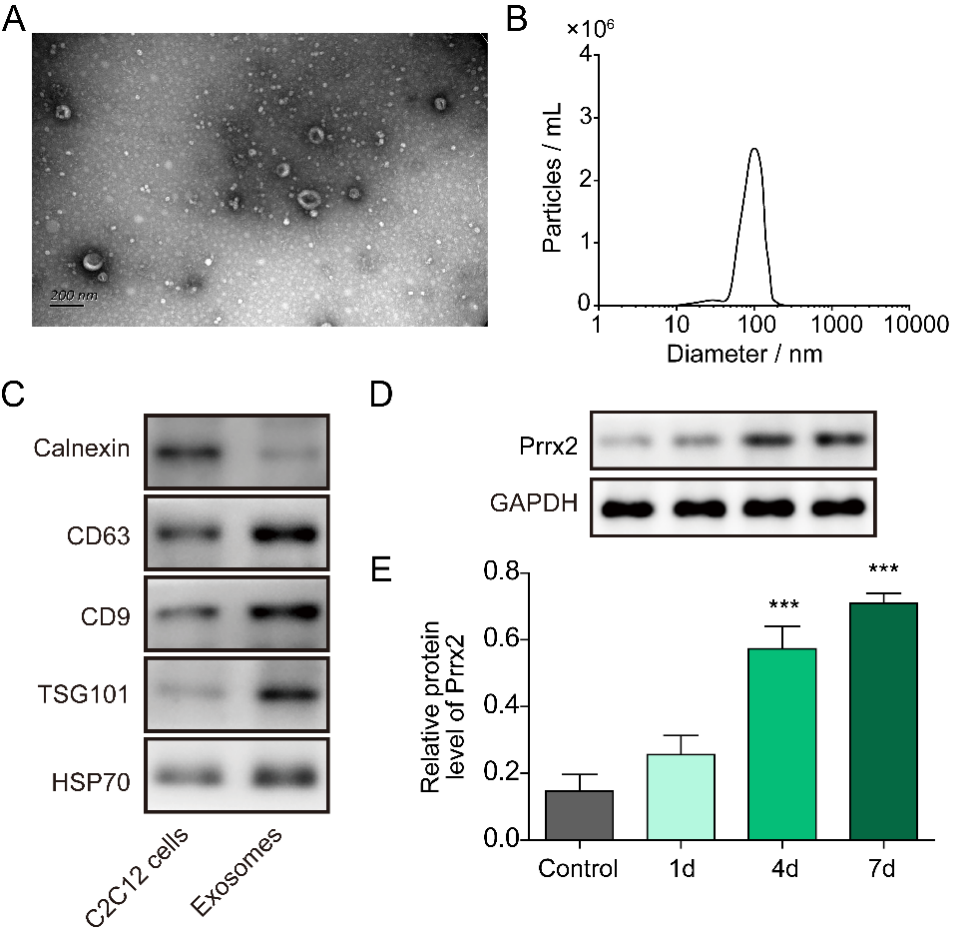
Up-regulation of Prrx-2 in myoblast-derived exosomes. (A) C2C12 myoblasts-derived exosomes were observed by transmission electron microscopy. (B) The particle sizes of exosomes were analyzed. (C) Western blotting was performed to detect the expression of exosome markers CD9, CD63, TSG101, HSP70 or endoplasmic reticulum marker Calnexin. (D&E) Prrx-2 level in isolated exosomes was evaluated by Western blotting. *** *P* < 0.001

First, the morphological changes of C2C12 cells during myogenic differentiation were observed. With the extension of differentiation time, more C2C12 cells displayed a longer shape than control cells, suggesting the formation of elongated myoblasts (Fig. S1A). Accordingly, the expression of myogenic markers (MyoD, MyoG and MyHC) was increased in myogenic differentiated C2C12 cells (Fig. S1B-D and S1F&G). In line with the elevation in MyoD, MyoG and MyHC expression, we found that the mRNA and protein levels of Prrx-2 were up-regulated in differentiated C2C12 cells (Fig. S1E-G). Moreover, we isolated exosomes from the supernatant of myogenic differentiated C2C12 cells. As observed by transmission electron microscopy, the size of exosomes arranged from 30 to 150 nM (Fig. 1A&B). Additionally, the expression of exosomal marker proteins CD63, CD9, TSG101, and HSP70 was higher, while the expression of endoplasmic reticulum marker Calnexin was lower in exosomes as compared with that in C2C12 cells (Fig. 1C), suggesting that exosomes were successfully extracted. Western blotting analysis indicated that Prrx-2 was up-regulated in the exosomes derived from C2C12 cells during myogenic differentiation (Fig. 1D&E). Collectively, Prrx-2 level was enhanced in differentiated myoblast C2C12 cells and their exosomes.

### Exosomal Prrx-2 derived from myogenic differentiated C2C12 cells promotes osteogenic differentiation of BMSCs

**Fig. 2:**
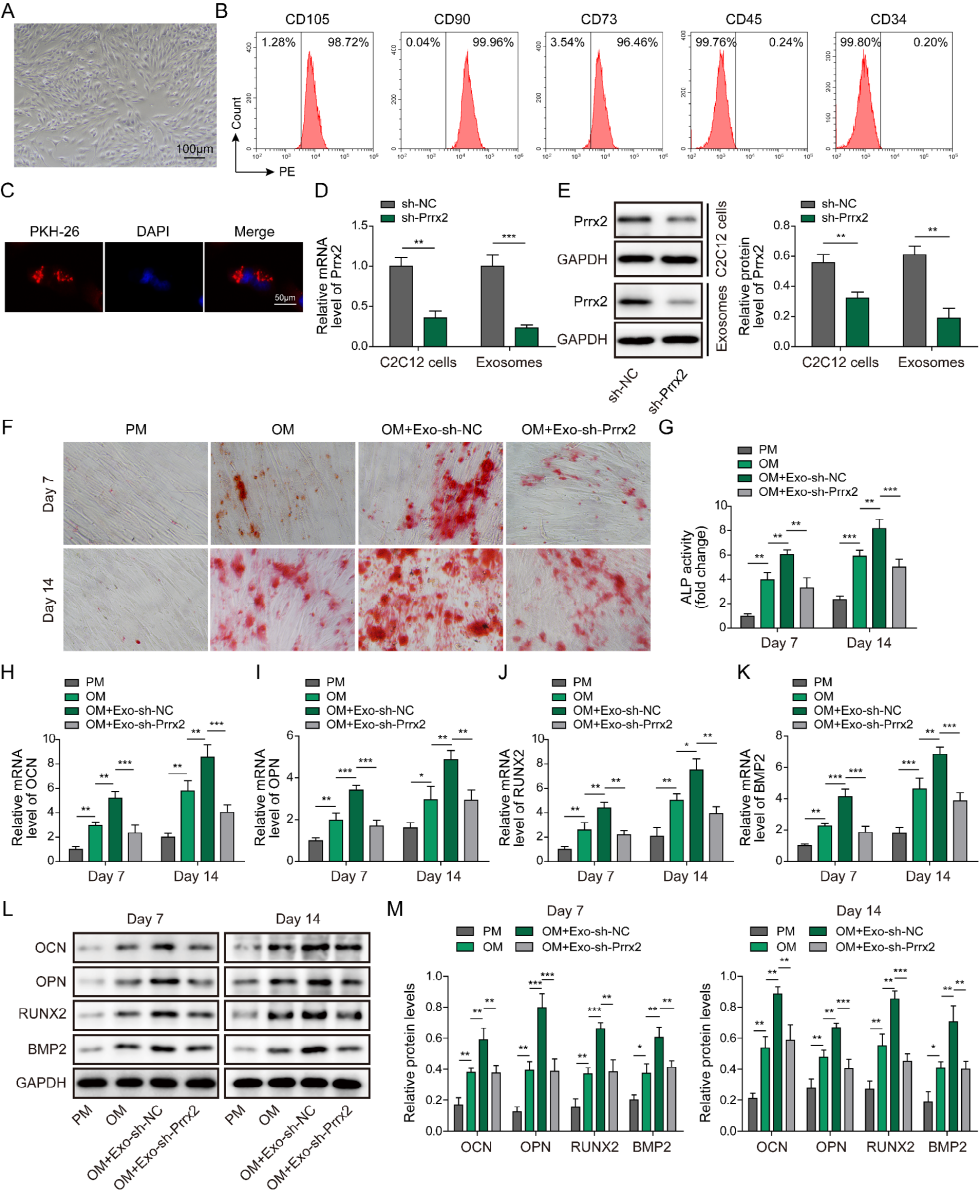
Myoblast-derived exosomal Prrx-2 facilitates osteogenic differentiation of BMSCs. (A) Morphology of isolated mouse BMSCs. (B) Flow cytometry confirmed the positive expression of CD105, CD90, CD73, and negative expression of CD45 and CD34 in BMSCs. (C) The internalization of PKH26-labeled exosomes by BMSCs was observed under a fluorescence microscopy. The expression of Prrx-2 in exosomes derived from Prrx-2-silenced C2C12 myoblasts was assessed by qRT-PCR (D) and Western blotting (E). (F) The calcium deposition of BMSCs was measured using Alizarin Red staining in the proliferation medium (PM), osteoblastic medium (OM), OM + Exo-shNC, OM + Exo-shPrrx-2 groups. (G) The ALP activity of BMSCs in the PM, OM, OM + Exo-shNC, OM + Exo-shPrrx-2 groups was determined. (H-M) qRT-PCR and Western blot analysis of the mRNA and protein levels of OCN, OPN, RUNX2, and BMP2 in the PM, OM, OM + Exo-shNC, OM + Exo-shPrrx-2 groups. * *P* < 0.05, ** *P* < 0.01 and *** *P* < 0.001

To explore whether exosomal Prrx-2 took part in osteogenic differentiation, BMSCs were isolated from mice. Figure 2 A shows the morphology of BMSCs. Furthermore, CD105, CD90, and CD73 were positively expressed, but CD45 and CD34 were negatively expressed in BMSCs (Fig. 2B), indicating a successful extraction of BMSCs. In addition, we observed the localization of PKH26-labelled exosomes in BMSCs, indicating the cellular uptake of exosomes by BMSCs (Fig. 2C). To investigate the biological function of Prrx-2, C2C12 cells were transfected with shPrrx-2. The knockdown efficiency of Prrx-2 was validated in C2C12 cells and exosomes (Fig. 2D&E). Alizarin red staining showed that incubation with OM for 7 d or 14 d remarkably facilitated osteoblast differentiation, which could be enhanced by C2C12 myoblasts-derived exosomes. Whereas incubation with exosomes derived from Prrx-2-depleted C2C12 myoblasts restrained osteoblast differentiation (Fig. 2F). Delivering exosomes further strengthened OM-induced increase in ALP activity, which was counteracted by exosomes isolated from Prrx-2-silenced C2C12 myoblasts (Fig. 2G). Moreover, incubation with OM led to increase in mRNA and protein expression of osteogenic differentiation-related markers (OCN, OPN, RUNX2, and BMP2) in BMSCs, which could be reinforced after treatment with C2C12 myoblasts-derived exosomes (Fig. 2H-M). However, incubation with exosomes derived from Prrx-2-silenced C2C12 myoblasts reversed the above results (Fig. 2H-M). These results revealed that inhibition of exosomal Prrx-2 derived from C2C12 myoblasts suppressed osteogenic differentiation of BMSCs.

### Myoblast-derived exosomal Prrx-2 enhances transcription and expression of MIR22HG in BMSCs via direct binding to MIR22HG promoter

**Fig. 3:**
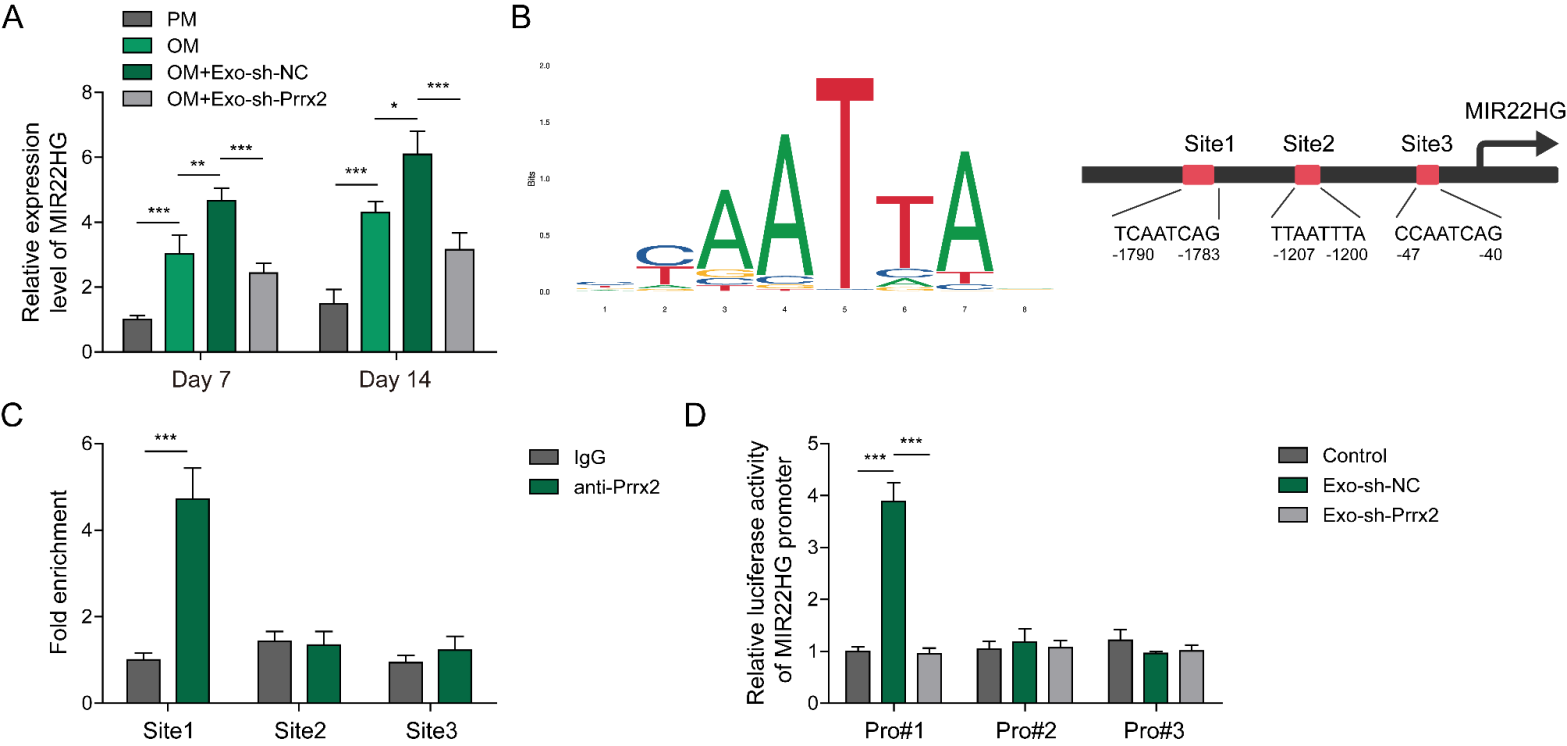
Myoblast-derived exosomal Prrx-2 increases transcriptional activity of lncRNA-MIR22HG in BMSCs. (A) qRT-PCR analysis of MIR22HG expression in BMSCs from the PM, OM, OM + Exo-shNC, OM + Exo-shPrrx-2 groups. (B) JASPAR database analysis of the binding sites of Prrx-2 in MIR22HG promoter. (C) ChIP and (D) Dual-luciferase reporter assays were adopted to validate the direct binding of Prrx-2 in MIR22HG promoter. * *P* < 0.05, ** *P* < 0.01 and *** *P* < 0.001

To gain insight into the mechanisms underlying myoblast-derived exosomal Prrx-2-mediated osteogenic differentiation, we focused on MIR22HG. As assessed by qRT-PCR, MIR22HG was up-regulated in osteogenic differentiated BMSCs, which was further promoted by C2C12 myoblasts-derived exosomes (Fig. 3A). However, treatment with exosomes derived from Prrx-2-silenced C2C12 myoblasts partly reversed the increased expression of MIR22HG (Fig. 3A). Furthermore, we predicted the interaction between Prrx-2 and MIR22HG promoter by using JASPAR database (Fig. 3B). ChIP assay demonstrated that Prrx-2 could bind to the binding site 1 in MIR22HG promoter, but not the binding site 2 or 3 (Fig. 3C). Additionally, the luciferase activity of MIR22HG promoter containing binding site 1 was enhanced by myoblast-derived exosomes, whereas silencing of Prrx-2 abolished the increased luciferase activity induced by exosomes (Fig. 3D). However, these changes were not observed in MIR22HG promoter containing binding site 2 or 3 (Fig. 3D). Therefore, myoblast-derived exosomal Prrx-2 promoted transcription of MIR22HG by directly binding to MIR22HG promoter in BMSCs.

### Myoblast-derived exosomal Prrx-2 inhibition restrains osteogenic differentiation via repressing MIR22HG expression

**Fig. 4:**
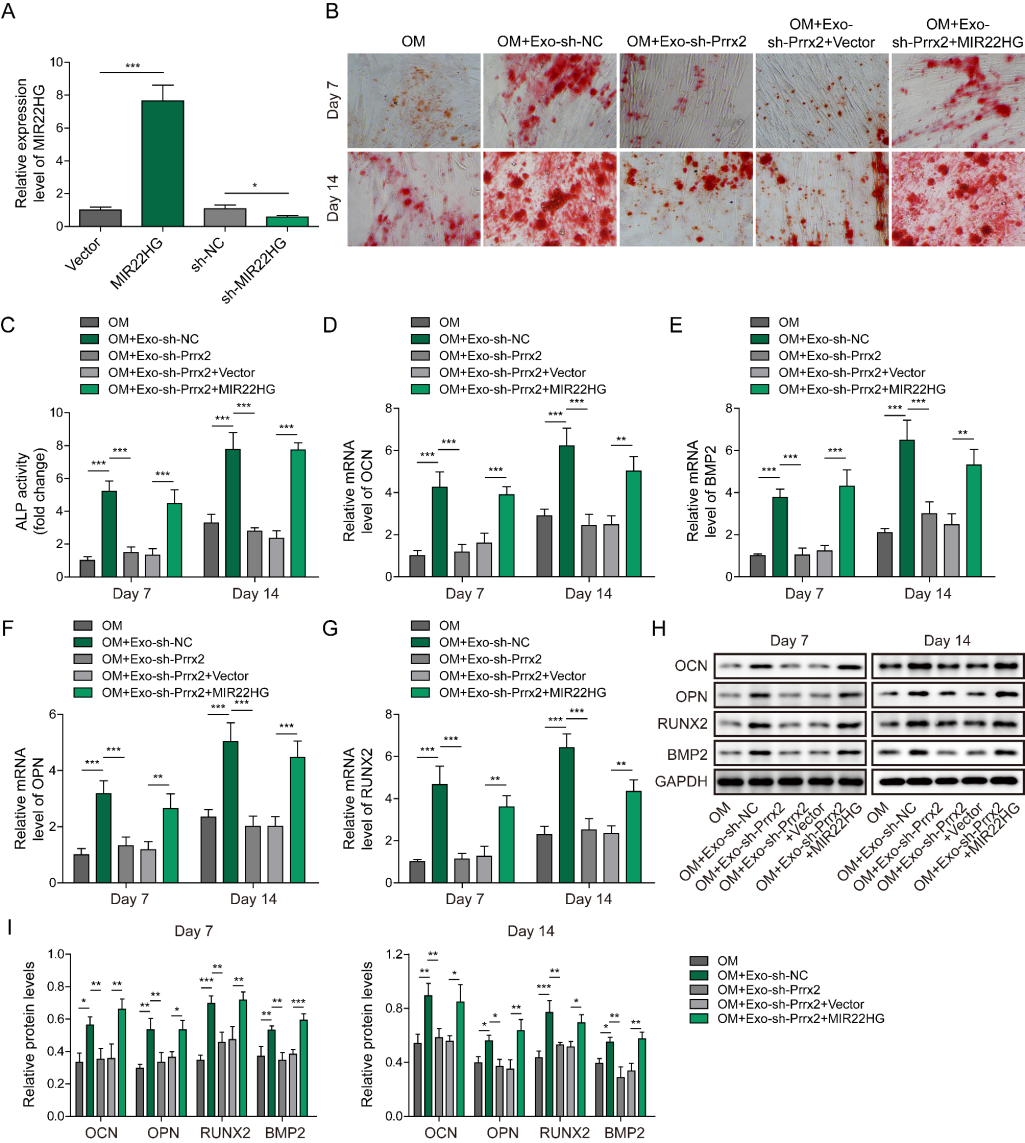
Silencing of Prrx-2 in myoblast-derived exosomes represses osteogenic differentiation via inhibiting MIR22HG expression. (A) MIR22HG expression in BMSCs fromVector, MIR22HG, shNC and shMIR22HG groups was assessed by qRT-PCR. (B) The matrix mineralization of BMSCs from OM, OM + Exo-shNC, OM + Exo-shPrrx-2, OM + Exo-shPrrx-2 + vector, OM + Exo-shPrrx-2 + MIR22HG groups was determined by Alizarin Red staining. (C) The ALP activity of BMSCs from different groups. (D-I) The mRNA and protein level of OCN, BMP2, OPN, and RUNX2 was analyzed by qRT-PCR and Western blotting. * *P* < 0.05, ** *P* < 0.01 and *** *P* < 0.001

To further validate the involvement of MIR22HG in myoblast-derived exosomal Prrx-2-mediated osteogenic differentiation, BMSCs were infected with lentiviruses containing MIR22HG overexpression plasmid or shMIR22HG. The overexpression or knockdown efficiency of MIR22HG in BMSCs was confirmed by qRT-PCR (Fig. 4A). The decreased matrix mineralization and ALP activity of BMSCs induced by inhibition of myoblast-derived exosomal Prrx-2 could be recovered by MIR22HG overexpression (Fig. 4B&C). Consistently, MIR22HG overexpression restored the decreased mRNA and protein levels of OCN, BMP2, OPN, and RUNX2 in BMSCs that were treated with exosomes derived from Prrx-2-silenced C2C12 myoblasts (Fig. 4D-I). The above observations suggested that depletion of myoblast-derived exosomal Prrx-2 inhibited osteogenic differentiation of BMSCs through directly restraining MIR22HG expression.

### MIR22HG activates YAP signaling pathway via targeting miR-128

**Fig. 5:**
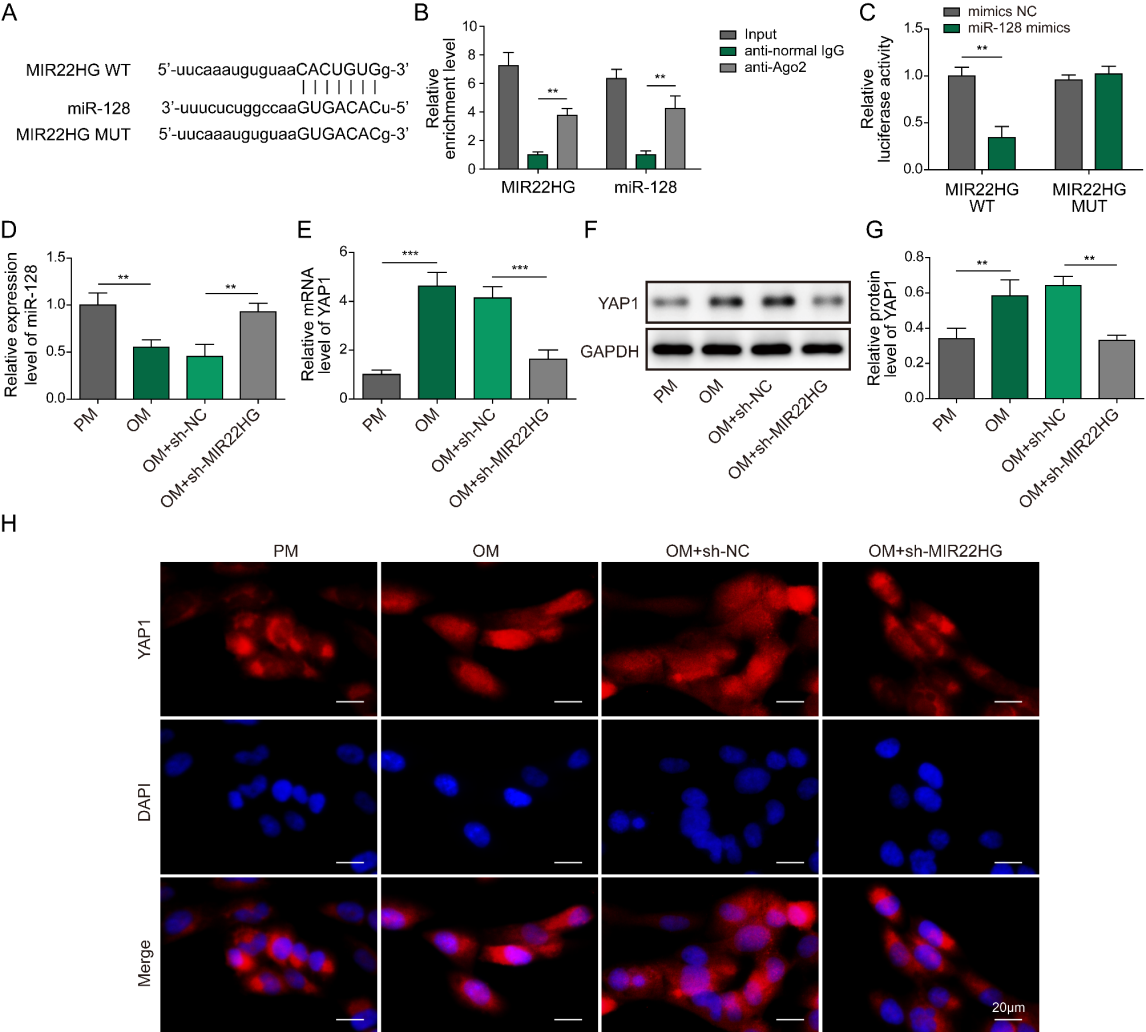
MIR22HG sponges miR-128 to activate YAP signaling pathway. (A) Starbase database predicted the binding sites of MIR22HG in miR-128. (B) RIP and (C) Dual-luciferase reporter assays confirmed the interaction between MIR22HG and miR-128. (D) miR-128 and (E) YAP1 mRNA levels in BMSCs from PM, OM, OM + shNC, OM + shMIR22HG were determined by qRT-PCR. (F&G) Western blotting analysis of YAP1 protein levels in BMSCs with various treatments. (H) The subcellular distribution of YAP1 in BMSCs was evaluated by immunofluorescence staining. ** *P* < 0.01 and *** *P* < 0.001

It is widely known that lncRNAs exert their functions via interacting with miRNAs, and lncRNA-miRNA-mRNA axis is involved in the regulation of osteogenic differentiation(Cai et al. 2020). To explore the downstream mechanism of MIR22HG in osteogenic differentiation, a potential target miRNA (miR-128) was predicted by Starbase database (Fig. 5A). RIP assay indicated that both MIR22HG and miR-128 could be enriched in AGO2 antibody-associated complex (Fig. 5B), indicating miR-128 was a MIR22HG-targeting miRNA. Dual luciferase reporter assay demonstrated that miR-128 mimics led to a declined luciferase activity of MIR22HG-WT, but not of MIR22HG-MUT (Fig. 5C), which further validated the direct interaction between MIR22HG and miR-128. Furthermore, we found that miR-128 level was declined in BMSCs cultured in OM, whereas knockdown of MIR22HG strikingly enhanced miR-128 level (Fig. 5D). In contrast, YAP1 mRNA level was increased during BMSC osteogenic differentiation, which could be reversed by MIR22HG silencing (Fig. 5E). Besides, the protein levels of YAP1 were up-regulated in osteogenic differentiated BMSCs, however, these changes were abolished in MIR22HG-depleted BMSCs (Fig. 5F&G). Immunofluorescence staining showed nuclear translocation of YAP1 during osteogenic differentiation, which was attenuated when MIR22HG was silenced (Fig. 5H). These data indicated that MIR22HG interacted with miR-128 to promote YAP1 expression, thereby activating YAP signaling pathway during BMSC osteogenic differentiation.

### MIR22HG depletion represses osteogenic differentiation via modulating miR-128

**Fig. 6:**
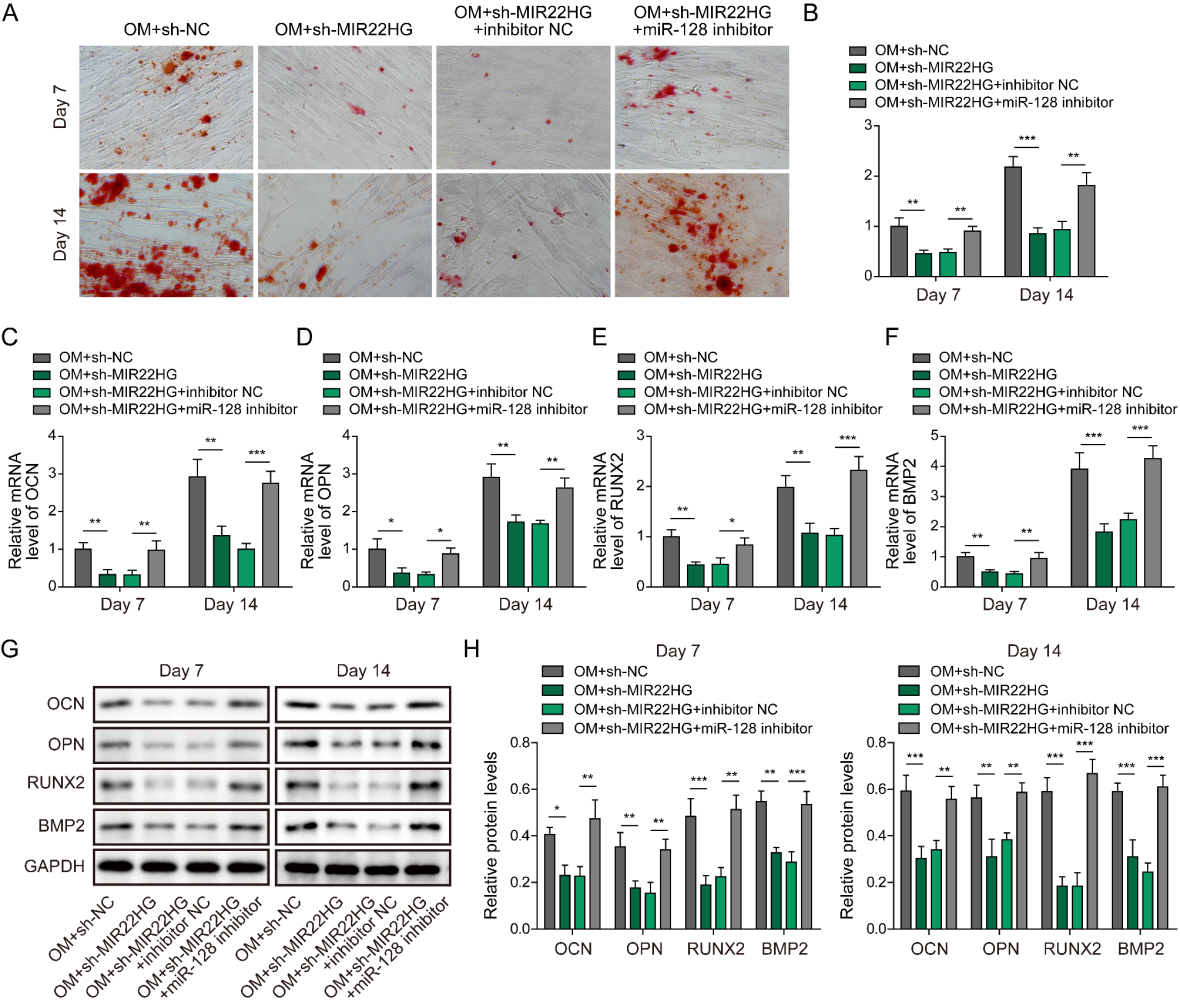
MIR22HG knockdown suppresses osteogenic differentiation via targeting miR-128. (A) Alizarin Red staining was carried out to assess the matrix mineralization of BMSCs with different treatments. (B) ALP activity of BMSCs was measured by commercial kit. (C-H) qRT-PCR and Western blot analysis of the mRNA and protein levels of OCN, BMP2, OPN, and RUNX2 in BMSCs. * *P* < 0.05, ** *P* < 0.01 and *** *P* < 0.001

To determine whether miR-128 was implicated in MIR22HG-mediated osteogenic differentiation regulation, MIR22HG-silenced BMSCs were further infected with lentiviruses encoding miR-128 inhibitor. We found that the matrix mineralization and ALP activity of BMSCs were reduced by MIR22HG knockdown, however, miR-128 inhibitor could counteract these changes (Fig. 6A&B). As presented in Fig. 6C-H, knockdown of MIR22HG lowered the mRNA and protein levels of OCN, BMP2, OPN, and RUNX2 at 7 d and 14 d after BMSC osteogenic differentiation, which were reversed by miR-128 inhibitor. Thus, MIR22HG silencing suppressed osteogenic differentiation of BMSCs via regulating miR-128.

### miR-128 inhibits Hippo signaling pathway by targeting YAP1 to decrease its expression and nuclear translocation

**Fig. 7:**
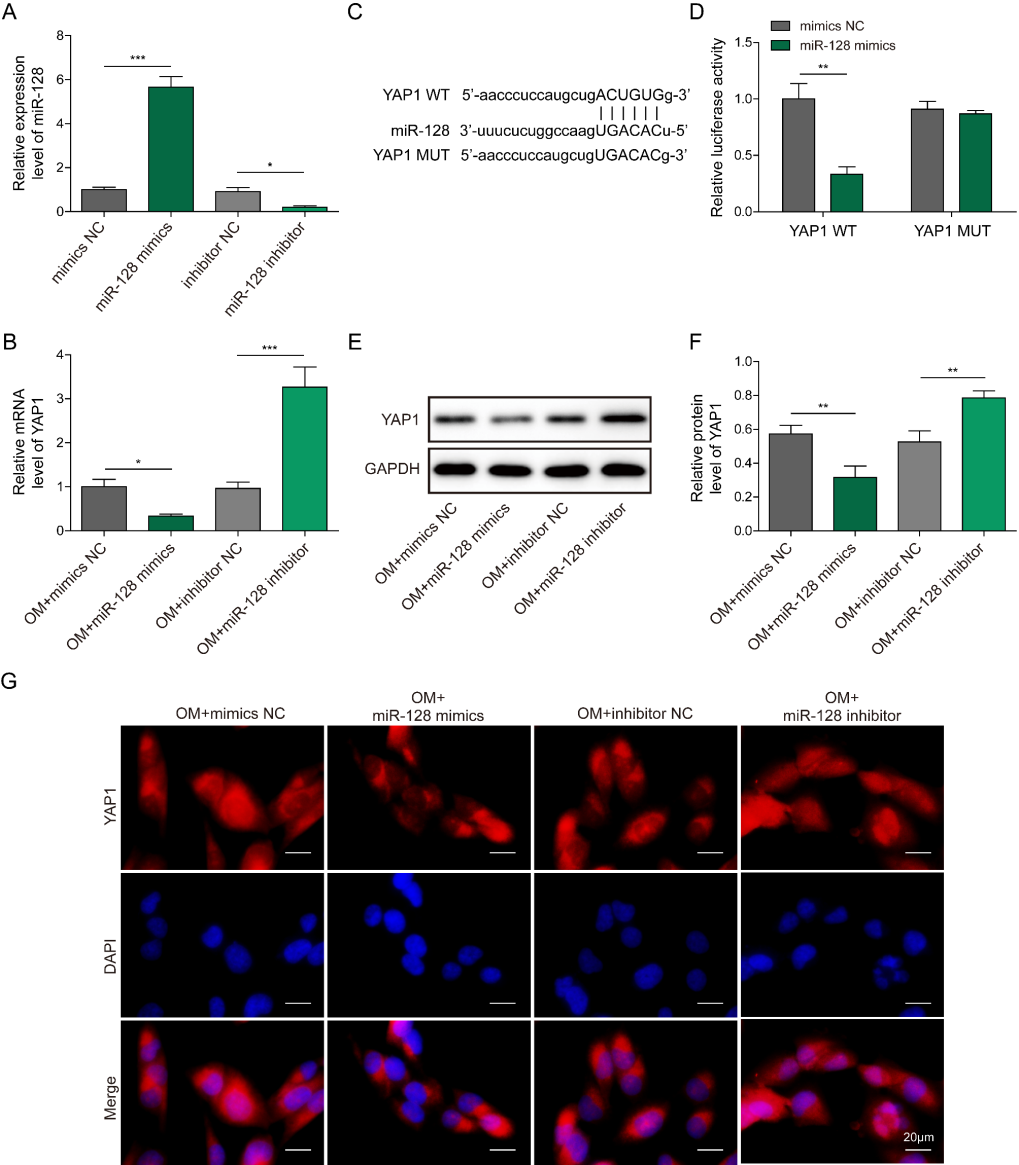
YAP1 is a downstream target gene of miR-128. (A) miR-128 and (B) YAP1 expression in BMSCs transfected with miR-128 mimics or miR-128 inhibitor was detected by qRT-PCR. (C) Starbase database was adopted to predict the binding sites of miR-128 to YAP1 3′UTR. (D) The interaction between miR-128 and YAP1 was analyzed by dual-luciferase reporter assay. (E&F) Western blotting analysis of YAP1 protein levels in BMSCs after transfection with miR-128 mimics or miR-128 inhibitor. (G) Immunofluorescence staining analysis of the subcellular distribution of YAP1 in BMSCs. * *P* < 0.05, ** *P* < 0.01 and *** *P* < 0.001

Next, we identified the target gene of miR-128. qRT-PCR assay indicated that overexpression of miR-128 down-regulated YAP1, while inhibition of miR-128 up-regulated YAP1 in BMSCs (Fig. 7A&B). Moreover, the putative binding sites of miR-128 in YAP1 were predicted by Starbase database (Fig. 7C). Using luciferase reporter assay, miR-128 mimics remarkably reduced the luciferase activity from YAP1-WT group, rather than that from YAP1-MUT group (Fig. 7D). The protein levels of YAP1 in BMSCs were decreased by miR-128 mimics, but raised by miR-128 inhibitor (Fig. 7E&F). As illustrated in Fig. 7G, the nuclear translocation of YAP1 was restrained by miR-128 mimics, however, miR-128 inhibitor led to the opposite result. These results demonstrated that YAP1 was direct target of miR-128 in BMSCs, and miR-128 suppressed Hippo signaling pathway via decreasing YAP1 expression and nuclear translocation.

### MiR-128 targets YAP1 to suppress BMSC osteogenic differentiation

**Fig. 8:**
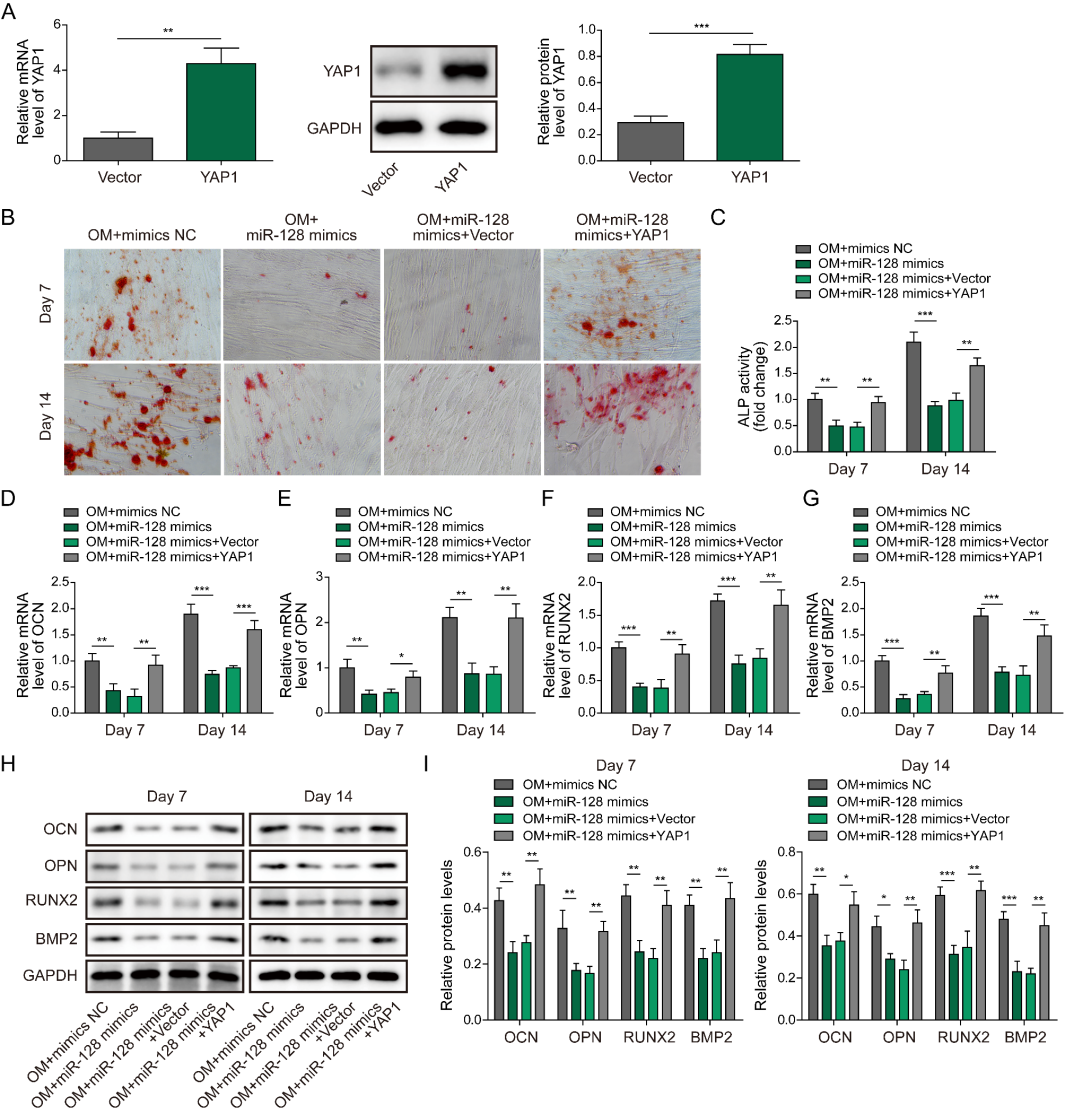
MiR-128 restrains BMSC osteogenic differentiation via targeting YAP1. (A) The overexpression efficiency of YAP1 in BMSCs was validated by qRT-PCR and Western blotting. (B) Alizarin Red staining was performed to detect the matrix mineralization of BMSCs from OM + mimics NC, OM + miR-128 mimics, OM + miR-128 mimics + vector, OM + miR-128 mimics + YAP1 groups. (C) ALP activity of BMSCs from multiple groups. (D-H) The mRNA and protein levels of OCN, BMP2, OPN, and RUNX2 in BMSCs were assessed by qRT-PCR and Western blotting. * *P* < 0.05, ** *P* < 0.01 and *** *P* < 0.001

After identifying YAP1 as a direct target of miR-128, the function of miR-128/YAP1 axis in osteogenic differentiation was investigated. Lentiviruses-mediated overexpression of YAP1 in BMSCs was confirmed by qRT-PCR and Western blotting (Fig. 8A). MiR-128 mimics restrained osteogenic differentiation of BMSCs as evidenced by decreased matrix mineralization and ALP activity, which could be recovered by YAP1 overexpression (Fig. 8B&C). Moreover, miR-128 mimics-mediated reduction in mRNA and protein expression of OCN, BMP2, OPN, and RUNX2 was abolished by ectopic YAP1 expression (Fig. 8D-I). Collectively, miR-128 exerted inhibitory roles in osteogenic differentiation via targeting YAP1.

### Myoblast-derived exosomal Prrx-2 relieves osteoporosis via MIR22HG to activate Hippo signaling pathway

**Fig. 9:**
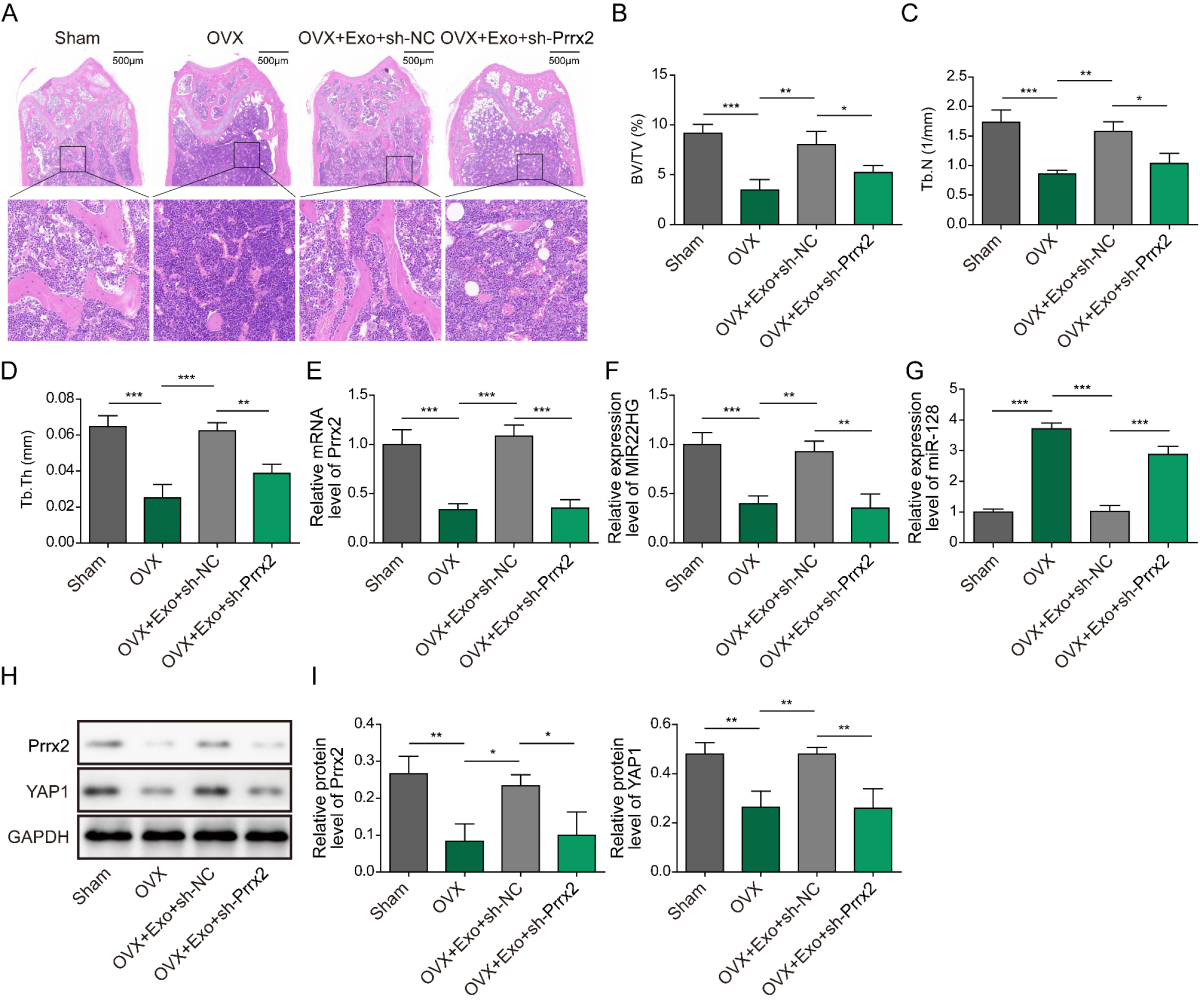
Delivering of myoblast-derived exosomal Prrx-2 represses bone loss via regulating MIR22HG/miR-128/YAP axis. (A) Decalcified femurs of mice were subjected to HE staining. (B-D) BV/TV, Tb.N, and Tb.Th were detected by micro-CT. (E-G) qRT-PCR analysis of Prrx-2, MIR22HG, and miR-128 expression levels in mouse femurs. (H&I) Western blotting analysis of Prrx-2 and YAP1 protein levels in mouse femurs. * *P* < 0.05, ** *P* < 0.01 and *** *P* < 0.001

To determine the effect of myoblast-derived exosomal Prrx-2 on osteoporosis in vivo, a mouse model of osteoporosis was established by OVX. HE staining revealed distinct bone loss in OVX mice. Treatment with myoblast-derived exosomes effectively attenuated OVX-induced bone loss, whereas depletion of Prrx-2 weakened the beneficial effect of myoblast-derived exosomes (Fig. 9A). Furthermore, micro-CT analysis showed that BV/TV, Tb.Th, and Tb.N were strikingly reduced in OVX group, as compared with sham group (Fig. 9B-D). Administration with myoblast-derived exosomes led to improvement of BV/TV, Tb.Th, and Tb.N levels, which could be reversed when Prrx-2 was knocked down (Fig. 9B-D). As determined by qRT-PCR, Prrx-2 and MIR22HG were down-regulated, while miR-128 was up-regulated in the femurs of OVX mice (Fig. 9E-G). With the treatment of myoblast-derived exosomes in osteoporotic mice, Prrx-2 and MIR22HG expression was increased, but miR-128 expression was decreased (Fig. 9E-G). However, these results were not obtained after treatment with exosomes-derived from Prrx-2-depleted myoblasts (Fig. 9E-G). Similarly, the decreased protein levels of Prrx-2 and YAP1 in OVX mice could be reversed by myoblast-derived exosomes, which were abrogated by Prrx-2 silencing (Fig. 9H-I). To sum up, myoblast-derived exosomal Prrx-2 attenuated osteoporosis through enhancing expression of MIR22HG, and subsequent activation of Hippo pathway via miR-128.

## Discussion

Osteoporosis and sarcopenia are superimposed gerontal diseases, resulting in a potential high risk of fracture and a lower life quality among the elderly(Huo et al. 2015). Osteosarcopenia is a new identified disorder with the coincidence of osteoporosis and sarcopenia (Hirschfeld et al. 2017). During osteosarcopenia development, osteoporosis and sarcopenia synergistically lead to a poor health outcome (Inoue et al. 2021). Therefore, uncovering the pathogenesis of osteosarcopenia to identify effective intervention is of great significance. In this study, up-regulation of Prrx2 in the exosomes derived from myoblasts contributed to BMSC osteogenic differentiation in vitro and restrained OVX-induced osteoporosis in mice in vivo. Prrx2 was demonstrated to bind to MIR22HG and promote its transcription, which subsequently caused activation of YAP via sponging miR-128. Our findings provided theoretical basis for targeting Prrx2 as a therapeutic option for osteosarcopenia.

The interaction between muscle and skeleton plays crucial roles in bone remodeling (Herrmann et al. 2020). Except for physical forces, secreted products are responsible for signal transmission between muscle and bone (Li et al. 2019). It is well accepted that exosomes are important mediators to transport miRNAs, lipids and proteins during the crosstalk between multiple tissues (Mathivanan et al. 2010). The contents of exosomes derived from myoblasts include nucleic acids, as well as proteins that are involved in osteogenesis. For example, signal transduction protein IGF-binding protein 5 (IGFBP-5) was identified in C2C12 myoblast-derived exosomes(Guescini et al. 2010). IGFBP-5 has been demonstrated to induce local bone formation in mouse and rat models (Bauss et al. 2001) and enhance early osteogenic differentiation of human bone marrow-derived mesenchymal stem cells (Zhang Z. M. et al. 2021b). Besides, heat shock proteins (HSP) 25, 70, and 90 were found to be abundant in C2C12 myoblast-derived exosomes (Guescini et al. 2010), which are essential for osteogenic differentiation (Shimada et al. 2003; Zhang et al. 2018). In addition, delivering of muscle-derived exosomal miR-23a cluster could promote bone metabolism (Zhang et al. 2021a). MiR-27a-3p carried by exosomes from C2C12 myoblasts could facilitate pre-osteoblasts MC3T3-E1 differentiation to osteoblasts (Xu et al. 2018). Prrx2 is a transcription factor necessary for skeletogenesis (ten Berge et al. 2001). Consistent with these observations, our findings demonstrated that Prrx2 expression was enhanced during myogenic differentiation, and muscle-derived exosomes carrying Prrx2 facilitated osteogenic differentiation. Our results uncover a novel molecule that contributes to the interaction between muscle and bone formation.

A series of studies have reported that transcription factors could regulate lncRNA expression in the process of osteogenic differentiation. Ye et al., documented that IRF2 improved the transcriptional activation of lncRNA-HHAS1 and facilitated osteogenesis (Ye et al. 2021). In periodontal ligament stem cells, E2F5 interacted with lncRNA-PCAT1 and formed a regulatory network by targeting miR-106a-5p, which enhanced osteogenic differentiation capacity (Jia et al. 2019). LncRNAs are pivotal regulators of bone metabolism and the involvement of lncRNAs in osteoporosis aetiology has been verified (Li et al. 2020; Wang et al. 2020). LncRNA-MIR22HG was found to be up-regulated during BMSC osteogenic differentiation, which favored osteogenesis in osteoporotic mice (Jin et al. 2020). However, the up-stream regulatory mechanisms of MIR22HG in osteogenesis are still unclear. In this work, myoblast-derived exosomal Prrx2 was demonstrated to bind to MIR22HG promoter and promoted MIR22HG expression, which facilitated osteogenic differentiation. Our findings firstly suggested that myoblast-derived exosomal Prrx-2 promoted osteogenic differentiation via enhancing transcriptional activity of MIR22HG.

Recently, a ceRNA theory has been described, suggesting that lncRNAs may function as miRNA sponges, thus competitively modulating the target genes of miRNAs (Tay et al. 2014). Mounting evidence has indicated that lncRNAs absorbed miRNAs to affect the expression of osteogenic differentiation-related genes in osteoporosis progression. Yu et al., documented that lncRNA RP11-84C13.1 led to improvement in capability of osteogenic differentiation by enhancing RUNX2 expression via sequestering miR-23b-3p (Yu et al. 2021). A recent study showed that miR-128 was highly expressed in osteoporotic patients, enforced expression of miR-128 suppressed osteoblast differentiation via targeting SIRT6 (Zhao et al. 2019). Besides, miR-128 up-regulation promoted osteoclastogenesis and was responsible for estrogen deficiency-induced osteoporosis (Shen et al. 2020). In the current study, miR-128 was demonstrated to be a target of MIR22HG. Moreover, inhibition of miR-128 counteracted the inhibitory effect of sh-MIR22HG on osteoblast differentiation. These findings suggested that increased expression of MIR22HG facilitated osteogenic differentiation via absorbing miR-128.

YAP1, a key downstream effector protein of the Hippo pathway, plays pivotal roles in various biological processes (Zhao et al. 2010). TAZ, the paralog of YAP1, is also a downstream effector of Hippo signaling (Sudol 1994). Activation of Hippo pathway may result in phosphorylation of TAZ, and inhibit YAP1 nuclear translocation (Deng et al. 2018). Particularly, the involvement of YAP1 in osteoblast differentiation has been verified. For instance, overexpression of YAP1 contributed to differentiation of mesenchymal stem cells into osteocytes (Guo et al. 2018). Pan et al., showed that YAP1 enhanced bone formation through modulating β-catenin pathway (Pan et al. 2018). It has been recognized that miRNAs modulate multiple biological processes by targeting mRNAs and silencing their expression (Zhu et al. 2017). Interestingly, we identified YAP1 as a downstream target of miR-128. miR-128 mimics reduced YAP1 level and restrained nuclear translocation of YAP1, whereas miR-128 inhibitor exhibited the opposite results. Additionally, the suppressive roles of miR-128 in BMSC osteogenic differentiation was abolished by YAP1 overexpression. Therefore, our data elucidated the participation of MIR22HG/miR-128/YAP1 axis in myoblast-derived exosomal Prrx-2-meidated osteogenic differentiation.

## Conclusions

Taken together, Prrx-2 was up-regulated in myogenic differentiated-C2C12 cells. Delivering of myoblast-derived Prrx-2 via exosomes enhanced BMSC osteogenic differentiation and alleviated OVX-induced bone loss via transcriptional activation of MIR22HG and subsequent increase in YAP1 expression by sponging miR-128. Our findings shed light on the potential mechanism of the interaction between muscle and skeleton, which provide a promising intervention for osteosarcopenia.

**Supplementary Fig. 1** Prrx-2 is up-regulated during myogenic differentiation of C2C12 cells. (A) Morphology of C2C12 cells after myogenic differentiation for 1 d, 4 d, and 7 d. qRT-PCR analysis of MyoD (B), MyoG (C), MyHC (D) and Prrx-2 (E) mRNA expression. (F&G) Western blotting analysis of MyoD, MyoG, MyHC and Prrx-2 protein levels. ** *P* < 0.01 and *** *P* < 0.001.

**Table:** 

Genes	Primer sequences (5’-3’)
MyoD	F: 5’-CGACACCGCCTACTACAGTG-3’
	R: 5’-GGTGGTGCATCTGCCAAAAG-3’
MyoG	F: 5’-CAGCGCCATCCAGTACATTG-3’
	R: 5’-CGCGAGCAAATGATCTCCTG-3’
MyHC	F: 5’-ACGCACCCTCACTTTGTACGC-3’
	R: 5’-CTCTGCCGAAAGTCCCCATAG-3’
Prrx2	F: 5’-ACGTGTCCAAGTCTGGTTCC-3’
	R: 5’-ATCTGGGCTCATCGTGGTAG-3’
OCN	F: 5’-AATCCAACGGTGTGAAGAGC-3’
	R: 5’-CCTGCGTGGAGTATCCATCT-3’
OPN	F: 5’-GCTTGGCTTATGGACTGAGG-3’
	R: 5’-TGGTTCATCCAGCTGACTTG-3’
RUNX2	F: 5’-AGATGGGACTGTGGTTACCG-3’
	R: 5’-GGACCGTCCACTGTCACTTT3’
BMP2	F: 5’-GGACCCGCTGTCTTCTAGTG-3’
	R: 5’-GTCTCTGCTTCAGGCCAAAC-3’
MIR22HG	F: 5’-GGACAGGTCGCAGTGATTTT-3’
	R: 5’-GCAGAGGGCAACAGTTCTTC-3’
miR-128	F: 5’-GCCGAGTCACAGTGAACCGGT-3’
	R: 5’-GTCGTATCCAGTGCAGGGTCCGA GGTATTCGCACTGGATACGACAAAGAG-3’
YAP1	F: 5’-TTCGGCAGGCAATACGGAAT-3’
	R: 5’-GTTGAGGAAGTCGTCTGGGG-3’
GAPDH	F: 5’-AGCCCAAGATGCCCTTCAGT3’
	R: 5’-CCGTGTTCCTACCCCCAATG-3’
U6	F: 5’-CTCGCTTCGGCAGCACA-3’
	R: 5’-AACGCTTCACGAATTTGCGT-3’

## Electronic supplementary material

Below is the link to the electronic supplementary material.

Supplementary Material 1

Supplementary Material 2

## Data Availability

Not applicable.

## Abbreviations

Prrx2: paired-related homeobox 2
lncRNAs: long non-coding RNAs
MIR22HG: MIR22 host gene
BMSCs: bone marrow mesenchymal stem cells
PM: proliferation medium
OM: osteoblastic medium
shNC: negative control shRNA
qRT-PCR: quantitative reverse transcription-polymerase chain reaction
ChIP: chromatin immunoprecipitation
WT: wild type
MUT: mutant
RIP: RNA immunoprecipitation
DAPI: 4′,6-diamidino-2-phenylindole
OVX: ovariectomized
BV/TV: bone volume per tissue volume
Tb.N: trabecular number
Tb.Th: trabecular thickness
HE: hematoxylin/eosin
YAP1: yes-associated protein 1
SD: standard deviation
