# Supplementary figures and images for: Myoblast-derived exosomal Prrx2 attenuates osteoporosis via transcriptional regulation of lncRNA-MIR22HG to activate Hippo pathway
Source: Mol Med. 2023 Apr 20;29:54. doi: 10.1186/s10020-023-00649-y (PMC10116833; doi:10.1186/s10020-023-00649-y)

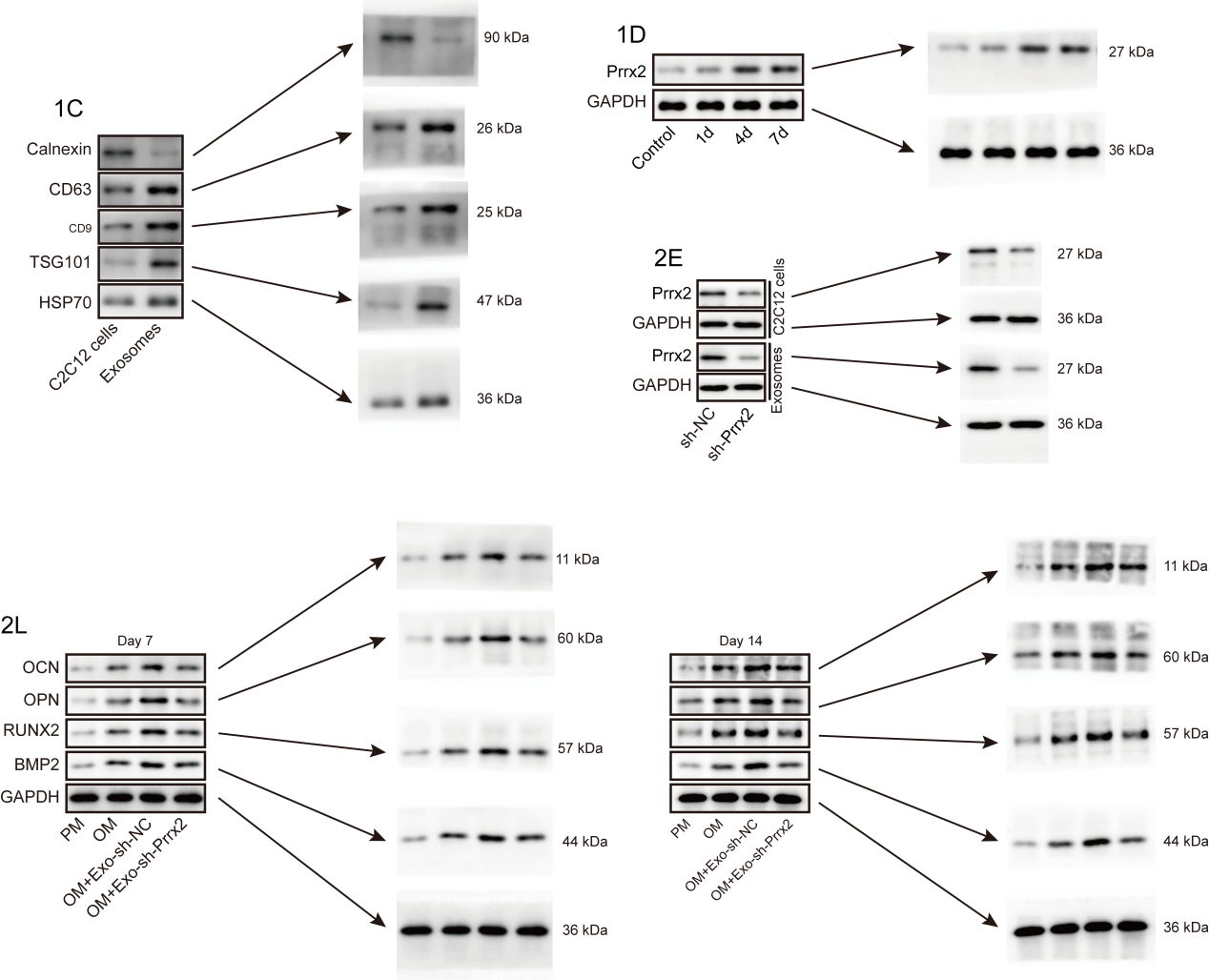


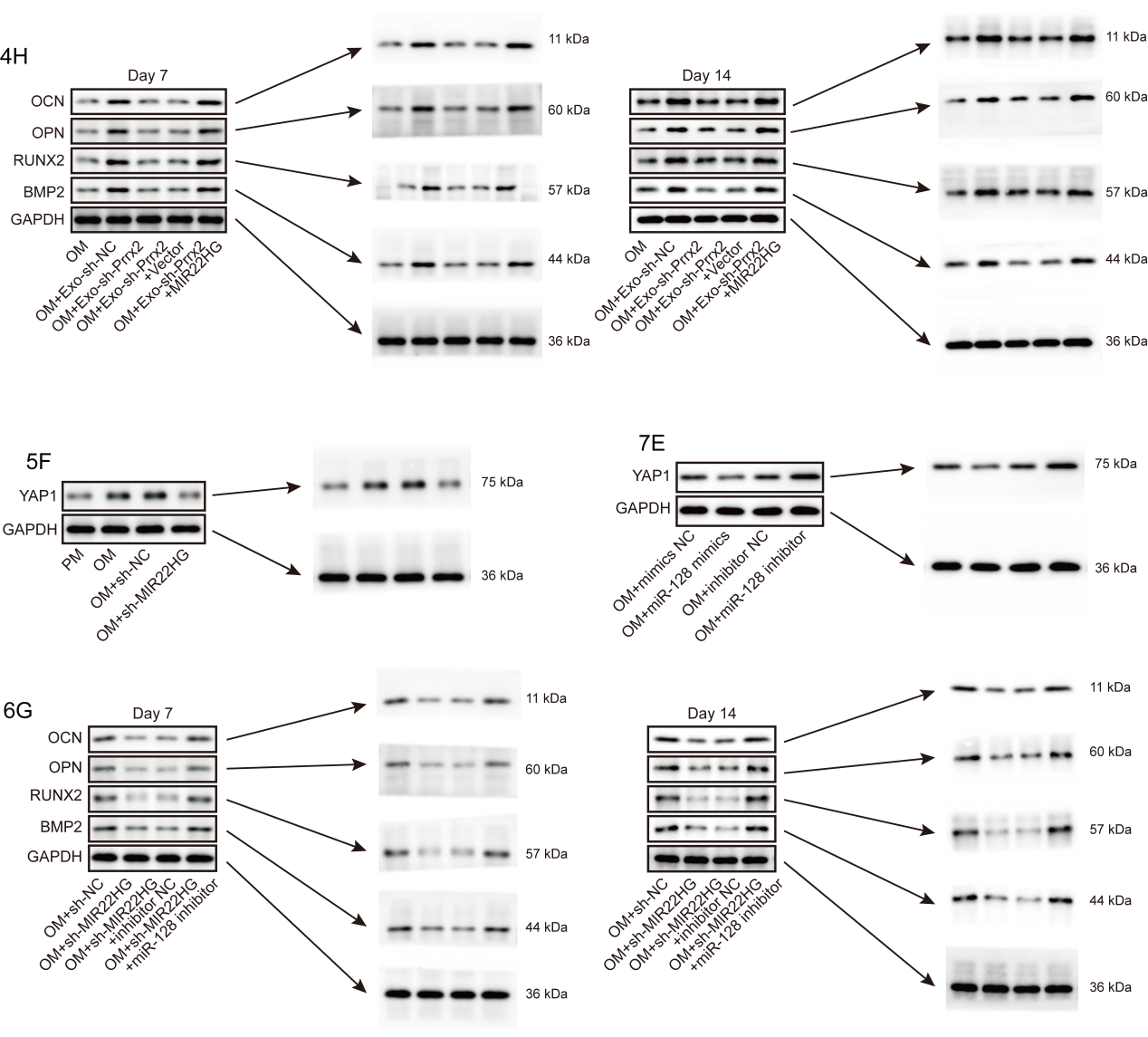


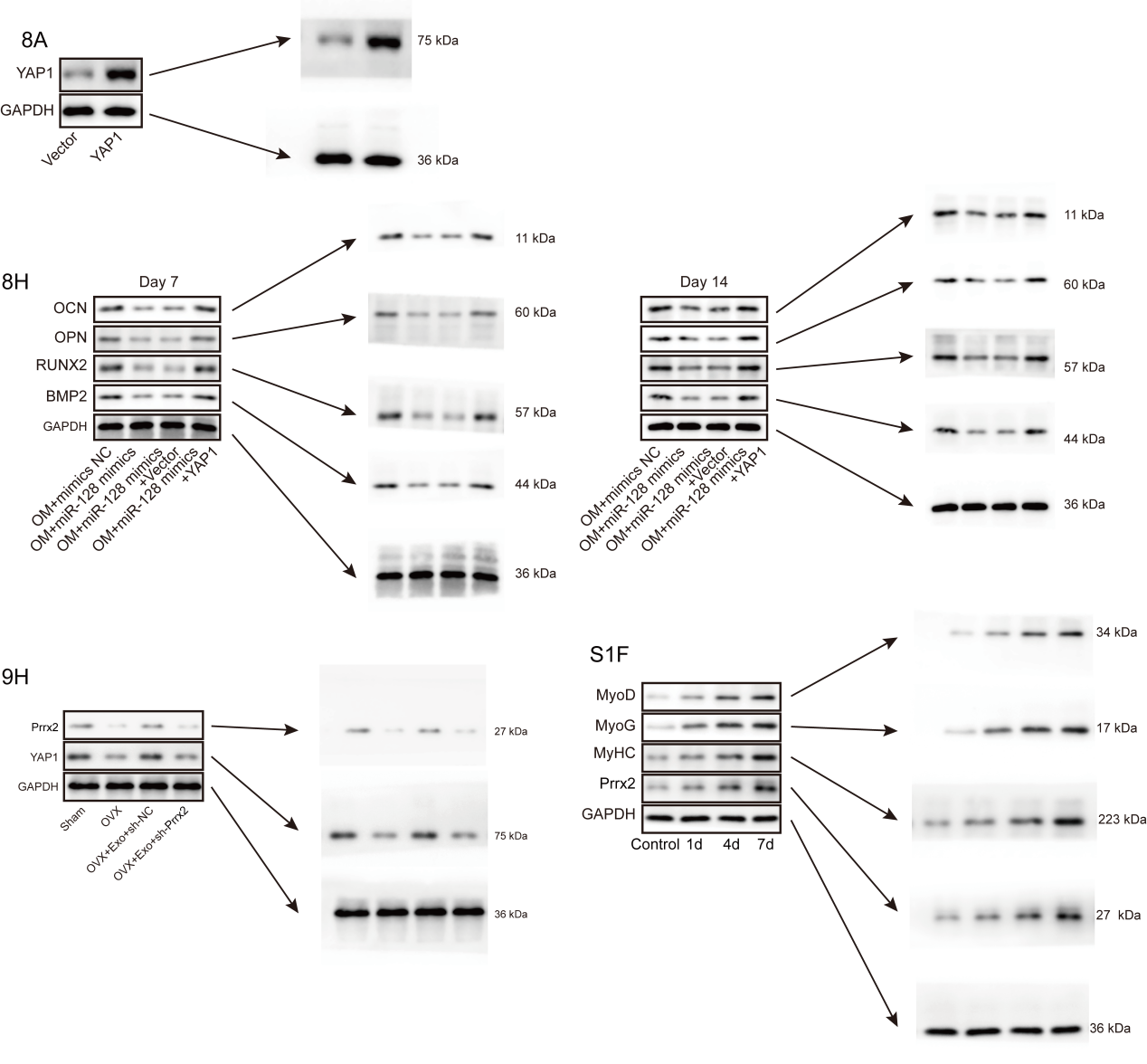

Supplement: Supplementary file 1 — Supplementary Material 1 [file 10020_2023_649_MOESM1_ESM.docx]

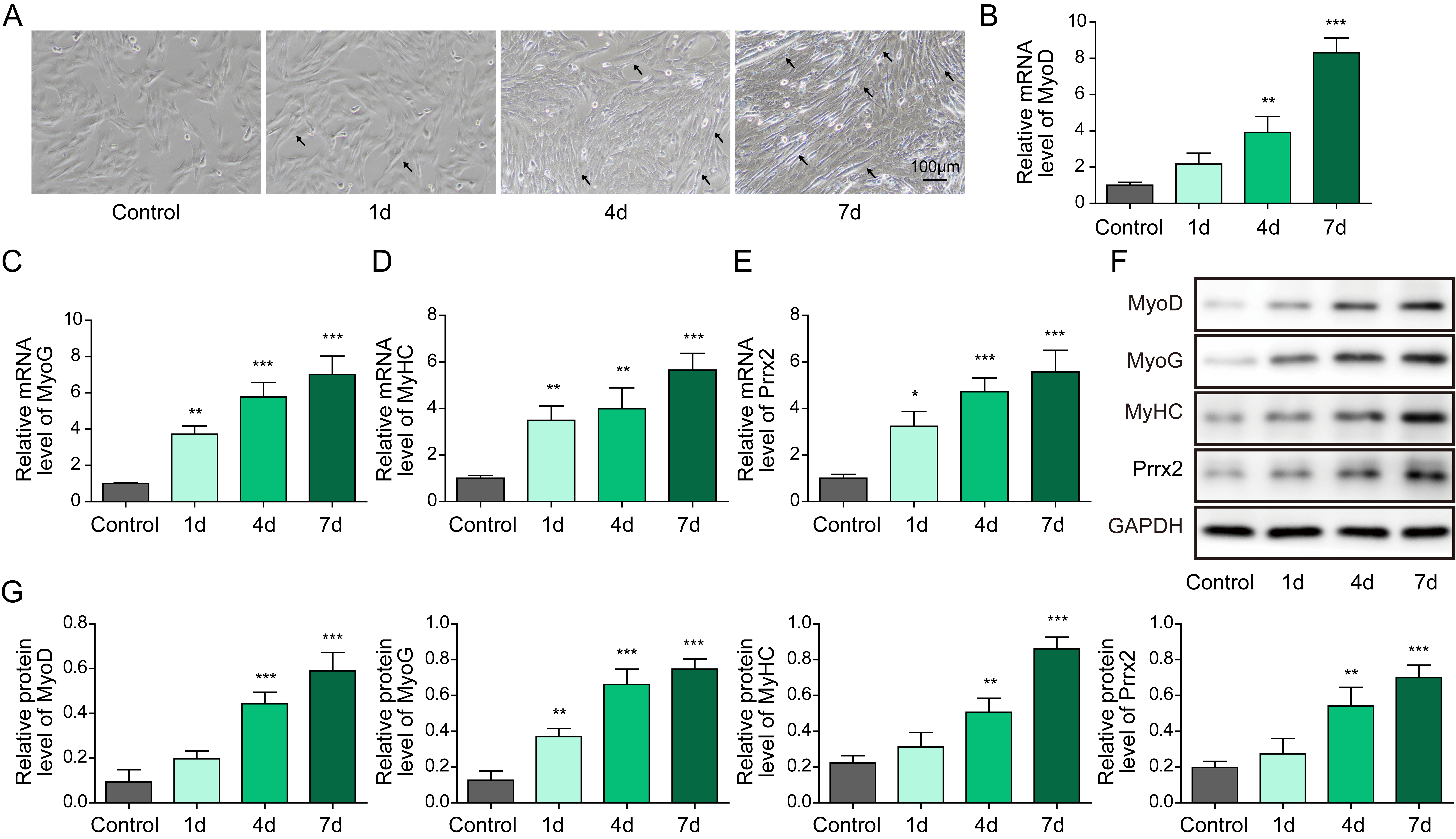

Supplement: Supplementary file 2 — Supplementary Material 2 [file 10020_2023_649_MOESM2_ESM.png]
